# Zoopharmacognosy in Diseased Laboratory Mice: Conflicting Evidence

**DOI:** 10.1371/journal.pone.0100684

**Published:** 2014-06-23

**Authors:** Minesh Kapadia, Hui Zhao, Donglai Ma, Rupal Hatkar, Monica Marchese, Boris Sakic

**Affiliations:** 1 Department of Psychiatry and Behavioural Neurosciences, McMaster University, Hamilton, Ontario, Canada; 2 Department of Pathology and Molecular Medicine, McMaster University, Hamilton, Ontario, Canada; 3 Department of Clinical Epidemiology and Biostatistics, McMaster University, Hamilton, Ontario, Canada; Radboud University, Netherlands

## Abstract

Zoopharmacognosy denotes a constellation of learned ingestive responses that promote healing and survival of infected or poisoned animals. A similar self-medication phenomenon was reported in diseased laboratory rodents. In particular, a series of studies revealed that autoimmune MRL/lpr mice readily consume solutions paired or laced with cyclophosphamide (CY), an immunosuppressive drug that prevents inflammatory damage to internal organs. However, due to design limitations, it could not be elucidated whether such a response reflects the learned therapeutic effect of CY, or a deficit in sensory input. We presently assess the behavioural effects of prolonged consumption of CY-laced, 16% sucrose solution in a continuous choice paradigm, with tap water available *ad lib*. Contrary to overall expectation, MRL/lpr mice did not increase their intake of CY with disease progression. Moreover, they ingested lower doses of CY and preferred less CY-laced sucrose solution than age-matched controls. The results obtained could not confirm zoopharmacognosy in diseased MRL/lpr mice, likely due to impaired responsiveness to palatable stimulation, or attenuated survival mechanisms after prolonged inbreeding in captivity. However, by revealing the effectiveness of unrestricted drinking of drug-laced sucrose solution on behavior and immunity, the current study supports broader use of such an administration route in behavioural studies sensitive to external stressors.

## Introduction

The term zoopharmacognosy is derived from Greek words *zoo* (animal), *pharma* (drug), and *gnosy* (knowing), denoting an evolutionary phenomenon comprised of mostly ingestive behaviors aimed at preventing or reducing the harmful effects of pathogens and toxins. Particularly, when sick, certain wild animals will ingest herbs, soil, dirt, or insects that have medicinal properties [1]. Great apes often consume plants with non-nutritional values that have beneficial effects on gut acidity [2], [3], or combat intestinal parasitic infection [4]–[6]. Similarly, domestic horses, dogs, cats, and cows infected with a pathogen may ingest herbs containing nutrients and chemicals with anti-parasitic properties [7]. More interestingly, cattle and some birds eat clay-rich termite mound soil, which deactivates ingested pathogens or fruit toxins [8], [9]. Although the underlying psychological and physiological mechanisms of such learned self-medicating behaviour are unclear, its adaptive value is proposed to be widespread [10], encompassing laboratory animals.

The MRL/MpJ-Fas^lpr^/2J (MRL/lpr) murine substrain spontaneously develops systemic autoimmune disease with clinical and serological manifestations reminiscent of human systemic lupus erythematosus, SLE [11]. Due to a *lpr* mutation on chromosome 19 and dysfunctional Fas receptor in negative selection of autoreactive T cells [12], [13], MRL/lpr mice develop florid disease by 3 months, with few surviving beyond 6 months of age [14]. Age-matched congenic MRL/MpJ +/+ (MRL +/+) controls develop a mild form of the disease and have a life span of up to two years [15]. Three studies by Grota and colleagues suggested that MRL/lpr mice adopt a learning pattern similar to wild animals to restore homeostasis within the immune system. Initial evidence came from a conditioning study, which utilized repeated pairings of access to a palatable stimulus (undiluted chocolate milk) with injections of a noxious, immunosuppressive drug cyclophosphamide (CY) after 23-hr fluid deprivation. Compared to MRL +/+ controls which manifest a mild form of disease, severely sick MRL/lpr mice did not show strong taste aversion learning when exposed to the 1-hr “chocolate milk vs. tap water” preference test [16]. This “learning deficit” was not observed prior to the development of systemic disease, or when non-immunosuppressive unconditioned stimuli were used, suggesting MRL/lpr mice rather associated chocolate milk with relief of autoimmune symptoms following pairings with CY. In an extension of their work, the same group demonstrated that fluid-deprived MRL/lpr mice “voluntarily” consumed more CY dissolved in chocolate milk than age-matched congenic MRL +/+ controls [17]. Consistent with the results from the conditioning study, consumption of CY solution differed between the two MRL substrains only when severe autoimmune symptoms in MRL/lpr mice emerged. Considering that dilutions of chocolate milk and fluid deprivation did not have a significant effect on CY consumption, it was proposed that autoimmune mice learned to consume more CY in an effort to correct their immune system dysregulation [18].

Although some classical conditioning studies have shown that rats can develop a preference for flavoured solutions associated with recovery from illness [19], [20], several lines of reasoning preclude the aforementioned inference on the perception of therapeutic effect and induction of “self-medication” in the studies above. Namely, impaired taste avoidance in conditioned autoimmune MRL/lpr mice [16] could be confounded by deficits that have been subsequently reported in this substrain including altered gustatory input [21], increased perseverance [22], and/or altered emotional reactivity and motivation [23]. As in the original taste aversion experiments, the study documenting “voluntary” consumption of CY-laced chocolate milk included 23-hr fluid deprivation [17], which introduces confounding effects of physiological and psychological stress induced by prolonged thirst. Moreover, repeated weekly bleedings from the retro-orbital sinus and anesthesia could also affect recovery and ingestive behaviour in the two groups. In the third study, where mice were fluid non-deprived, mean volume of CY-laced chocolate milk consumed on weekdays was merely ∼1 ml higher in a large cohort of diseased MRL/lpr mice (N = 86) when compared to age-matched controls, N = 78 [18]. Although intake was compared between two substrains and genders, no comparisons were made between mice exposed to CY and vehicle only, or shown as a preference over plain water intake, which was available during weekends only. In summary, such an invasive, single-choice, cross-sectional approach precludes a clear answer on whether increased CY consumption in diseased mice is due to differences in thirst levels, responsiveness to a palatable solution/protein content, or indeed – to an altered immune status. If the therapeutic effect of CY is perceived and an adaptive behavioural strategy is learned, one may expect increased preference for a therapeutic drug as the autoimmune disease progresses. Using a continuous choice paradigm and *ad lib* access to tap water, we presently examine this possibility by longitudinally assessing the preference for CY-laced sucrose solution in non-deprived, age-matched MRL/lpr and MRL +/+ mice. A more appropriate design may employ aqueous CY solution, thus eliminating the caloric and incentive properties of sucrose. However, due to its unpalatable, “metallic” taste and negligible intake of CY dissolved in water [18], we mixed CY with 16% sucrose solution to avoid protein content, increase palatability and thus achieve the therapeutic dose range of CY (∼70–100 mg/b.w.). Functional effects of voluntary CY and sucrose intake were assessed using a battery of behavioural tests and immunopathological measures.

## Methods

### Animals

Thirty 8 week old MRL/lpr (stock 485) and 30 age-matched MRL/MpJ (MRL +/+, stock 486) male mice were purchased from the Jackson Laboratories (Bar Harbour, ME). Upon arrival, they were tail-tattooed for identification purposes (AIMS Inc., Hornell, NY) and housed in groups of 5 mice/cage under standard laboratory conditions (light period from 7∶00 A.M. –7∶00 P.M., food and water *ad libitum*). Starting at 11 weeks of age, mice were individually housed 3 days per week for assessment of ingestive behaviour and body weight. Two MRL +/+ mice were excluded from the study due to excessive inter-male aggression. Loss >20% of body mass, necrotic ear tips, and severe alopecia/dermatitis were exclusion criteria and signs for early euthanasia. Three MRL/lpr mice were sacrificed due to disease manifestations, thus reducing the sample size to N = 55. All procedures were approved by the local Animal Care Committee (McMaster University AUP #11-03-11) and performed in accordance to guidelines set out by the Canadian Council of Animal Care.

### Immunosuppressive Treatment

Mice were assigned into four groups (*n* = 15 mice/group), according to substrain (MRL/lpr vs. MRL +/+) and treatment (CY vs. vehicle, Veh). For a 12-week period, all mice had *ad lib* access to a top-mounted 10 ml plastic syringe containing CY (“Procytox”, Baxter, Mississauga, ON) diluted to 0.4 mg/ml in 16% sucrose solution, or Veh (16% aqueous solution of sucrose). The use of 16% sucrose as the vehicle was based on previous evidence that MRL substrains do not differ in sucrose intake in a 48-hr, one-bottle test [24]. The CY/sucrose solution was available over 3 days each week. Intake of water, sucrose and CY-laced sucrose solutions was recorded daily, when syringes were re-filled with fresh solution.

### Behavioural Battery

Behavioural data were collected between the 21^st^–22^nd^ weeks of age, or before overt manifestations of SLE-like disease (e.g., alopecia, dermatitis, necrotic ears) develop in the MRL/lpr group [11]. Anxiety-like response to an elevated space was assessed in the step-down test as the latency to descend from a raised platform onto a black cloth in a brightly-lit room [23]. Blunted responsiveness to sucrose solutions in a choice-paradigm is proposed to reflect impaired sensitivity to palatable stimulation, and as such, to model “anhedonia” [25]–[27]. A brief sucrose preference test was given on the days when water was available only, as described previously [24]. To examine the functional status of the hippocampus [28], spontaneous T-maze alternation was assessed using the discrete-trial procedure [29]. Muscle strength and sensorimotor coordination were evaluated using a Rota-Rod (ENV-575M, Med Associates Inc., St. Albans, VT), which accelerated from 4 to 40 RPM over the course of 5 min. The latency to fall in three daily trials was recorded. Increased immobility (floating) in the forced swim test was measured with EthoVision XT 8 software package (Noldus, NL) and defined as a <5% change in surface area between successive samples. In addition to the above behavioural battery, spontaneous behaviour in custom-made activity cages equipped with running wheels was monitored between 3∶00–5∶00 P.M. Live-tracking of animal locomotion was performed by EthoVision XT 8. After each session, mice were returned to their home-cage and activity boxes were cleaned with Quatricide disinfectant (1∶256 dilution, PRL Pharmacal, Naugatuck, CT).

### Immunopathology

At 22 weeks of age, mice were anaesthetized with a ketamine/xylazine mixture, exsanguinated by severing the inferior vena cava, and intracardially perfused with phosphate buffered saline. Brains were extracted and sera were stored at −20°C until further analysis. Extracted organs were weighed on an analytical balance (Mettler Toledo AB54-S; VWR Scientific of Canada, Mississauga, ON). Wet spleen mass was recorded to assess splenomegaly (enlarged spleen), a reliable indicator of autoimmune disease in lupus-prone mice [15]. In addition, serum levels of anti-dsDNA, anti-cardiolipin and anti-proteinase 3 (PR3) autoantibodies were quantified using a fully automated ELISA analyzer (EUROIMMUN Analyzer I). Briefly, 100 µl of each sample (serum 1∶50 dilution in sample buffer) was transferred into the corresponding microtiterplate well (EUROIMMUN pre-coated microtiterplate). For anti-dsDNA analysis, each well contained antigen substrate of dsDNA complexed with nucleosomes and coupled to the solid phase. Quantification of anti-cardiolipin antibodies was performed using wells coated with purified cardiolipin isolated from bovine heart. For assessment anti-PR3 antibodies, microplate wells were coated with a mixture of recombinant and native PR3. Irrelevant of the antibody tested, each sample was incubated for 30 min at room temperature and then washed three times with 450 µl of working strength wash buffer. One hundred microliters of 1∶2000 diluted rabbit anti-mouse IgG-HRP conjugate (Promega) was pipetted into each of the microtiterplate wells, left to incubate, and washed to remove unbound HRP enzyme conjugate. Subsequently, 100 µl of 3, 3, 5, 5 tetramethylbenzidine enzyme/substrate solution was pipetted into each well of the microtiterplate and incubated for another 20 min at room temperature. One hundred microliters of stop solution was added to each well in the same order and at the same speed as when the chromogen/substrate solution was introduced. The microtiterplate was shaken at a speed of 20 Hz for 5 s to ensure a homogeneous distribution of the solution. Optical density was determined at a wavelength of 450 nm and a reference wavelength of 620 nm within 10 min of adding the stop solution. Observed results are expressed as relative optical densities.

### Statistical Analysis

Normal distribution of the data was tested by the Shapiro-Wilk test. In cases when data departed from normality, the overall assumption was that parametric tests (such as ANOVA and ANOVA with repeated measures) were robust enough to detect significant group differences because the cohorts were independent, sample sizes were equal, and population variances were comparable, as revealed by Levine’s test. Analysis of variance (ANOVA) and Analysis of Covariance (ANCOVA) were used in overall analysis, with Substrain (MRL/lpr vs. MRL +/+) and Treatment (CY vs. Veh) as between-group factors. Age, Concentration or Trials were considered within-group factors in ANOVA with repeated measures. Student’s t-test was used in post-hoc comparisons. Considering a significant reduction in body weight was noted when habituation and the behavioural battery was applied (20–22 weeks of age), body weight was considered a covariate in ANCOVA when a significant correlation between behavioural performance and weight was observed. In instances involving non-homogeneity of variance or departure from normal distribution, nonparametric tests (e.g., Kruskal-Wallis, Mann-Whitney) were used. Pearson’s correlation was used to examine linear relationships between behavioural measures, body weight, and immunological parameters. Statistical significance was set at *p*≤.05 for all comparisons. Graphs indicate mean values and ± SEM with significant differences of *p*≤.05, *p*<.01 and *p*<.001, shown as *, **, and ***, respectively. All calculations were performed using SPSS 20 software package (SPSS Inc., Chicago, IL).

## Results

**Figure 1:**
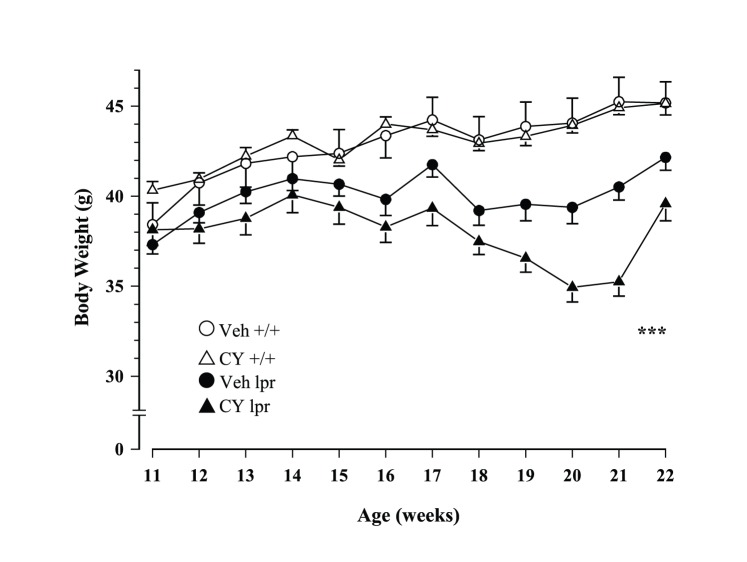
Mean body weight from 11–22 weeks of age. Autoimmune MRL/lpr mice were significantly lighter when compared to congenic controls. However, prolonged immunosuppression further reduced their body weight, but not in MRL +/+ mice.

In general, MRL/lpr males were lighter than controls throughout the study (Substrain: F_1,51_ = 25.461, p<.001), but prolonged exposure to CY further reduced their body weight at an older age (Substrain×Treatment×Age: F_11,561_ = 4.245, p<.001; **Figure 1**).

### 

#### 3.1. Ingestive behaviour

**Figure 2:**
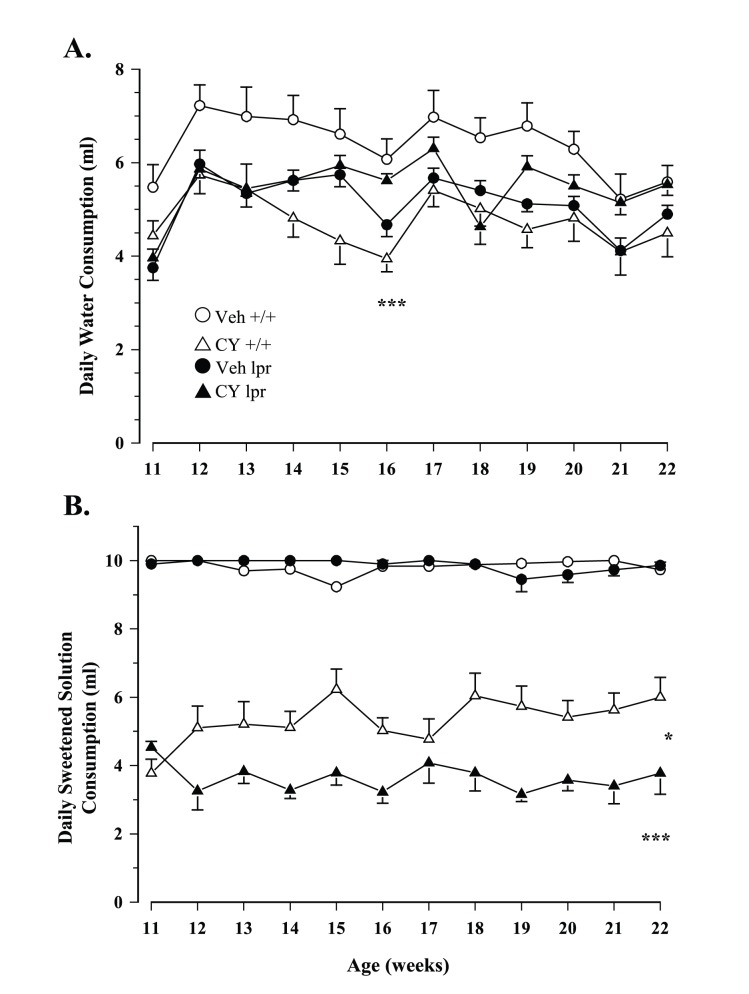
Daily fluid consumption from 11–22 weeks of age. (A) Despite fluctuations in daily water intake over the course of the study, Veh MRL +/+ mice consumed the most water in the 2-bottle test. They also drank significantly more than their CY +/+ counterparts, a trend that was not noted in the MRL/lpr substrain. (B) Lacing sweetened solution with CY significantly reduced daily consumption. However, autoimmune MRL/lpr mice consumed significantly less CY solution in comparison to MRL +/+ mice, particularly after the first week of treatment.

**Figure 3:**
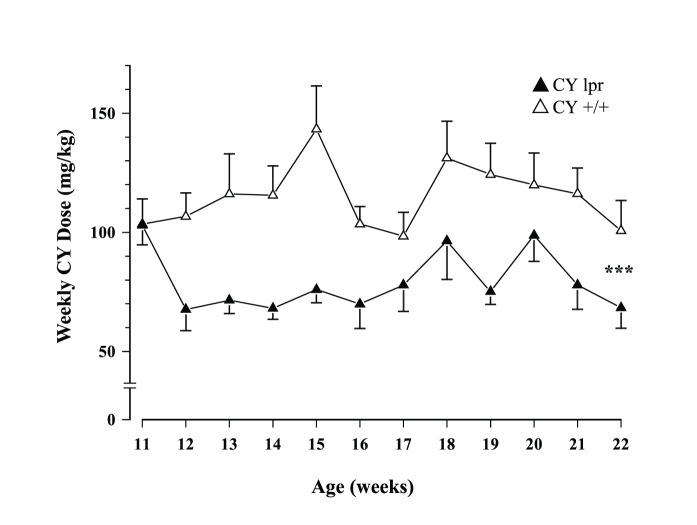
Weekly CY dose ingested. Although both substrains received similar CY dose at the starting of the treatment period, voluntary drinking in subsequent weeks was higher in less symptomatic MRL +/+ than in MRL/lpr mice.

**Figure 4:**
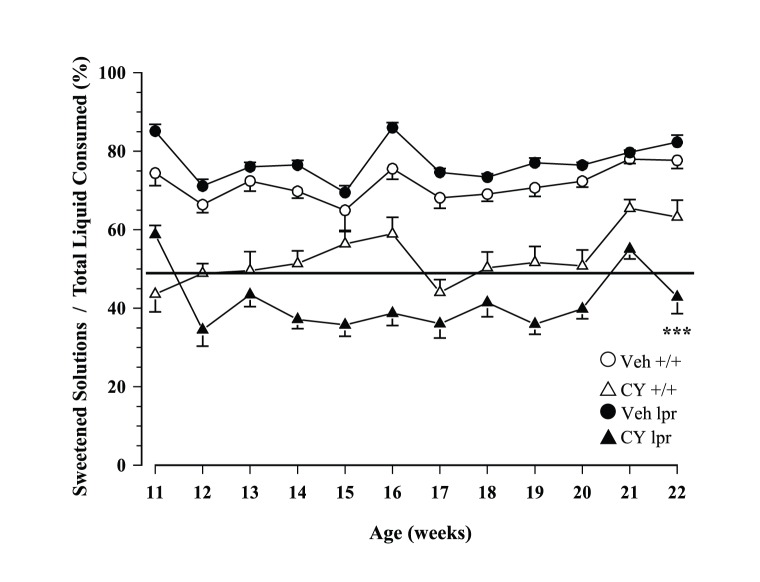
Weekly preference for sweet solutions. Initially, CY lpr mice displayed a higher preference for CY/sucrose in comparison to control CY +/+ group at 11 weeks of age. However, this difference was transient and became the lowest of all groups by the end of the study.

Total daily fluid intake ranged from ∼7–11 ml in young mice and declined to ∼7–9 ml when they got older (Age: F_1,51_ = 9.232, p<.001). Although MRL/lpr mice exposed to CY drank comparable volumes of water as the conspecifics exposed to sucrose, the CY +/+ control group displayed significantly lower intake than the Veh +/+ group (Substrain×Treatment: F_1,51_ = 13.666, p<.001; **Figure 2A**). When sucrose solutions were available, the presence of CY significantly reduced overall intake, particularly in the MRL/lpr group after the 11^th^ week of age (Treatment: F_1,51_ = 672.76, p<.001; Substrain×Treatment×Age: F_1,51_ = 3.253, p = .031; **Figure 2B**). Consequently, the weekly CY dose consumed via voluntary drinking was higher in less symptomatic MRL +/+ than in MRL/lpr mice with early disease manifestations (100–150 mg/kg in +/+ mice vs. 60–100 mg/kg b.w. in lpr mice; Substrain: F_1,27_ = 28.322, p< .001; **Figure 3**). Similar results were obtained when group differences in fluid consumption were offset by calculating preference as intake of sweetened solutions/total fluid intake. Although preference for CY/sucrose was higher in the CY lpr group than in the CY +/+ group at 11 weeks of age (U = 40, p<.005), it declined in the subsequent week and remained the lowest of all groups throughout the study (Substrain×Treatment×Age: F_11,561_ = 3.553, p<.001; **Figure 4**). Contrary to our expectation, their preference for CY was far below that of the diseased Veh lpr group, making these two groups even more dissimilar than the MRL +/+ groups (Substrain×Treatment: F_1,51_ = 24.614, p<.001).

#### Other behaviours

**Figure 5:**
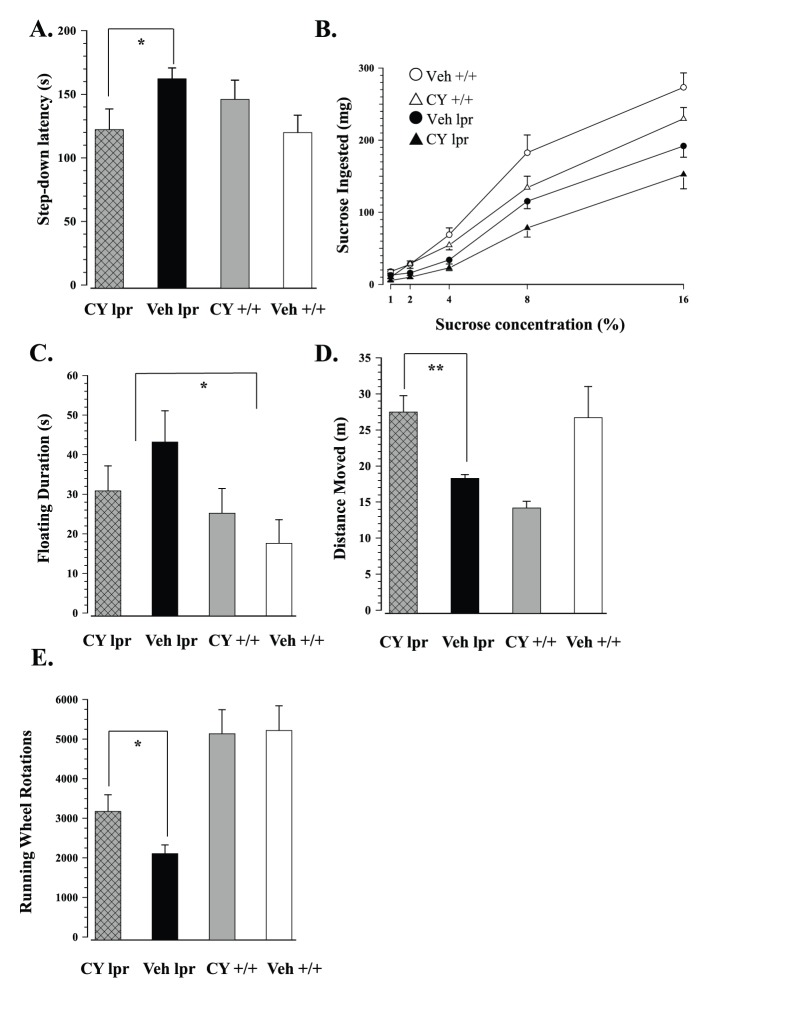
Behavioural effects of chronic exposure to CY/sucrose solution. (A) Chronic immunosuppression reduced step-down latency in diseased MRL/lpr mice, but had the opposite effect on congenic controls. (B) In addition to the previously-documented substrain differences in sucrose consumption [24], CY had a significant detrimental effect on the performance in both groups. (C) Although a trend was noted, CY could not abolish previously-reported differences in overall floating in the forced swim test [23]. (D) Prolonged immunosuppression improved spontaneous ambulatory activity in MRL/lpr mice, but had a detrimental effect on MRL +/+ controls. (E) Conversely, CY exposure significantly improved (but did not normalize) running wheel activity in MRL/lpr mice.

CY treatment significantly reduced step-down latency of MRL/lpr mice, but had the opposite effect on congenic controls (Substrain×Treatment: F_1,51_ = 5.582, p<.05, **Figure 5A**). Body weight at 21 weeks correlated positively with total sucrose intake in the brief preference test (r_55_ = 0.685, p<.001). Expectedly, a significant effect of body weight on performance was observed with ANCOVA (Weight: F_1,50_ = 8.094, p<.01). Nevertheless, sucrose intake in autoimmune MRL/lpr mice was generally lower than in control MRL +/+ mice (Substrain: F_1,50_ = 4.327, p<.05). Moreover, there was a trend for CY-treated mice to ingest less of the solution in comparison to the Veh groups (Treatment: F_1,50_ = 3.792, p = .057; **Figure 5B**).

Rota-Rod data revealed that CY exposure improved performance of MRL/lpr mice, but hindered that of MRL +/+ mice (Substrain×Treatment: F_1,51_ = 5.379, p<.05; data not shown). A significant negative correlation between body weight and time spent on the accelerating rod suggested that smaller mice performed better (Trial 1: r_52_ = −0.394, p<.01; Trail 2: r_52_ = −0.540, p<.001; Trial 3: r_52_ = −0.401, p<.01). Indeed, repeated measures ANCOVA with body weight as a covariate revealed a significant effect of body weight on the latency to fall (Weight: F_1,50_ = 9.370, p<.01), thus accounting for the above group difference (Substrain×Treatment: F_1,51_ = 3.409, n.s.).

With the present sample size, chronic CY treatment failed to normalize increased immobility in MRL/lpr mice (Substrain: F_1,50_ = 5.419, p = .024, **Figure 5C**). Moreover, no significant treatment effect was noted in other variables including distance swam, velocity, and overall swimming topography (data not shown). In spontaneous T-maze alternation, all groups performed comparably in both acquisition and reversal trials (data not shown), suggesting CY does not affect hippocampal-dependent spatial memory and learning in MRL substrains. Conversely, prolonged exposure to CY prevented the decline in spontaneous ambulatory activity in MRL/lpr mice, but had a detrimental effect on the control MRL +/+ group (Substrain×Treatment: F_1,42_ = 22.661, p<.001; **Figure 5D**). While CY was unable to abolish substrain difference in running wheel activity (Substrain: F_1,42_ = 27.113, p<.001; **Figure 5E**), it did improve the performance of treated MRL/lpr mice in comparison to the Veh lpr group (t_22_ = −2.255, p = .034).

### Immunopathology

**Figure 6:**
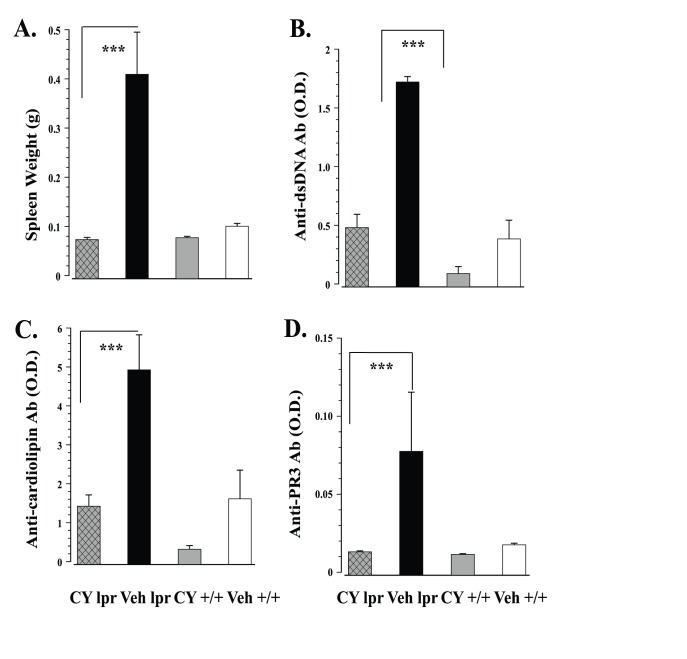
Immunosuppressive effects of continuous exposure to CY/sucrose solution. Generalized immunosuppression in CY-exposed MRL/lpr mice evidenced by normalized spleen weight (A), as well as significant reductions in serum levels of autoantibodies directed towards dsDNA (B), cardiolipin (C), and PR3 (D) antigens.

Chronic immunosuppressive treatment prevented splenomegaly in MRL/lpr mice (Substrain×Treatment: F_1,52_ = 11.350, p<.001, **Figure 6A**). Normalized spleen mass was accompanied by significantly lower serum anti-dsDNA (Substrain×Treatment: F_1,55_ = 20.933, p<.001, **Figure 6B**), anti-cardiolipin (Treatment: F_1,52_ = 29.001, p<.001, **Figure 6C**), and anti-PR3 autoantibody levels in this group (*K*_3_ = 37.141, p<.001, U = 8.0, P<0.001 **Figure 6D**). In summary, unrestricted exposure to sweetened CY solution abolished severe signs of systemic autoimmunity. However, it did not prevent well-documented low brain weight in the MRL/lpr substrain (data not shown) [30], [31].

## Discussion

The current study tested the hypothesis that zoopharmacognosy is operational in laboratory mice affected by a progressive, chronic disease. The overall premise was that when autoimmune/inflammatory disease manifests, MRL/lpr mice will increase preference for an immunosuppressive drug because of learned therapeutic consequences. Compared to studies which involved classical conditioning and fluid-deprivation [17], [18], we used a rather “ethological approach” by providing *ad lib* access to water and immunosuppressive CY solution. Such a design enabled experimental animals to freely choose between the solutions, akin to the unrestricted access to diverse fluids and foods in natural habitats available to wild mice. However, our results do not support self-medication in laboratory mice, because none of the three expectations were met. Firstly, MRL/lpr mice did not increase their consumption of CY-laced sucrose solution alongside disease progression. Secondly, they failed to display higher preference for CY-laced sucrose solution in comparison to diseased Veh lpr and asymptomatic CY +/+ controls. Lastly, the dose of CY ingested (a measure independent of the preference) was lower in diseased MRL/lpr mice than in CY +/+ group. Therefore, the notion that autoimmune mice consume solutions that have been paired or laced with CY in an “effort to correct their immune system dysregulation” [18] could not be confirmed with the present set of results. As such, this study is in line with a contrarian view on “dietary wisdom” in omnivores that was largely “attributed both to overreliance on theory and to lack of critical attention to data” [32].

If a therapeutic effect of an immunosuppressant can be perceived and learned indeed, one may expect that diseased MRL/lpr mice would drink CY dissolved in water. However, this was not observed by Grota and colleagues, prompting the authors to dilute CY in chocolate milk to improve its palatability [18]. In the current study, we counteracted the taste of CY with 16% sucrose solution and observed comparable CY consumption in the first week of exposure (Figure 3). This confirmed that taste sensitivity to CY was comparable in 11 week-old MRL/lpr mice and age-matched MRL +/+ controls. Subsequent decrease in intake/preference of the CY lpr group however, does not seem to reflect nutritional or learning deficit. This conclusion is based on evidence that diseased MRL/lpr mice show preference for carbohydrate-rich food [e.g., Veh lpr group performance, Figure 4; [33]] and do not display generalized learning deficit [16], [34]. Therefore, one may assume that reduced preference for CY-laced sucrose mixture and impaired performance in the brief sucrose preference test in both drug-treated groups are attributable to the repelling and cytotoxic properties of CY. Moreover, it is documented that even acute exposure to CY reduces the number of taste buds and impairs responsiveness to sucrose in mice [35], [36]. In the MRL/lpr strain however, severe systemic disease *per se* may further lead to reduced gustatory nerve responses [21] and depressive-like behaviour [23], [24], which may jointly aggravate their response to sucrose in the present study.

The current findings are also in line with the study testing the notion of “dietary wisdom” in diabetic rodents [37]. Specifically, laboratory rats affected by a diabetes-like disease failed to develop a strong preference for high fat diets, proposed to have beneficial post-ingestional properties. In the MRL strain, one may hypothesize that altered genetic make-up after repeated inbreeding impaired the adaptive survival mechanisms operational in wild animals. In support of this hypothesis, inbreeding has been shown to have significant detrimental effects on the survivorship of mice reintroduced into a natural habitat [38], offspring survival and reproductive success [39]. Indeed, the effects of laboratory inbreeding can be exemplified by the gradual decline in the autoimmune phenotype *(* http://jaxmice.jax.org/strain/006825.html *)* and severity of behavioral deficits in the MRL/ lpr substrain [40], [41].

Although zoopharmacognosy could not be shown in the present study, two other important concepts emerged from the data collected. The first is the causal link between autoimmunity and aberrant behaviour. CY proved to be a potent immunosuppressant that abolished systemic immunopathology, as shown by reduced spleen weight and autoantibody levels in MRL/lpr mice. These findings are consistent with previous observations that brief, 1-hr access to CY-laced chocolate milk over 3–4 weeks results in significant reductions in lymphadenopathy and serum anti-ssDNA antibody levels [17], [18], as well as with generalized immunosuppression after repeated 100 mg/kg/week intraperitoneal CY injections [42]. Along the same line, *ad lib* access to the immunosuppressive solution abolished several behavioural deficits, as shown previously for anxiety-like and motivated behaviours following CY treatment [24], [42]. Conversely, the lack of effectiveness in preventing excessive floating in the forced swim test and brain atrophy suggest a role for non-immunological factors, such as genetic lesions and/or neuroendocrine changes [43].

The second concept that emerged relates to the non-invasive administration route of a noxious drug in experimental mice. Indeed, the present results suggest that lacing it with a palatable ingredient can produce a therapeutic dose comparable to the injection route. As shown in Figure 3, the weekly dose voluntarily ingested was relatively constant in each substrain and can likely be adjusted by increasing the number of exposure days and/or concentration of sucrose or the drug itself. By avoiding repeated exposure to handling, oral gavage, and injections, such an approach can minimize the confounding effects of stress and reduce variance, thus increasing consistency and precision across behavioural and physiological studies.
